# Intestinal microbiota regulates anti‐tumor effect of disulfiram combined with Cu^2+^ in a mice model

**DOI:** 10.1002/cam4.3346

**Published:** 2020-08-04

**Authors:** Hong Hu, Lanyue Cui, Jiachen Lu, Kehong Wei, Jing Wei, Shaobo Li, Changwei Zou, Tingtao Chen

**Affiliations:** ^1^ School of Life Sciences Nanchang University Nanchang PR China; ^2^ National Engineering Research Center for Bioengineering Drugs and the Technologies Institute of Translational Medicine Nanchang University Nanchang PR China; ^3^ School of Resources Environmental and Chemical Engineering Key Laboratory of Poyang Lake Environment and Resource Utilization Ministry of Education Nanchang University Nanchang China

**Keywords:** disulfiram, gut microbiota, inflammatory cytokine, melanoma xenograft

## Abstract

**Background:**

A growing number of studies show that intestinal microbiota affect the therapeutic effects of antineoplastic agents. Disulfiram (tetraethylthiuram disulfide, DSF) is an old alcohol‐aversion drug that has been shown to be effective against various types of cancers in preclinical studies, while few studies are carried out to explore its mechanism.

**Methods:**

A mice model of melanoma xenograft was generated and treated with antibiotics (Abx), disulfiram/copper (DSF/Cu^2+^), Abx + DSF/Cu^2+^, and the tumor volume and survival curve were observed. Hematoxylin‐eosin (HE) staining and western blotting (WB) were used to observe the protein changes related to cell morphology, inflammation, and apoptosis in tumor tissues. Quantitative real time polymerase chain reaction (qPCR) was used to detect the expression of pro‐inflammatory cytokines in tumors. High‐throughput sequencing was used to detect the effects of Abx and DSF/Cu^2+^ on intestinal microbiota.

**Results:**

The DSF/Cu^2+^ and Abx + DSF/Cu^2+^ markedly delayed tumor progression and prolonged mice survival, of which the combination of Abx and DSF/Cu^2+^ possessed the best anti‐tumor effect. Abx + DSF/Cu^2+^ significantly reduced the pro‐inflammatory cytokines Interleukin‐1β (IL‐1β), IL‐6 and tumor necrosis factor α (TNF‐α) in tumors, and significantly reduced the expression of phosphorylated‐protein kinase B (p‐AKT)/protein kinase B (AKT), toll‐like receptors 4 (TLR‐4), and phosphorylated‐ nuclear factor kappa‐B (p‐NFκB)/NFκB in tumors. Moreover our high‐throughput sequencing first indicated that the sound anti‐cancer effect of Abx + DSF/Cu^2+^ had a strong connection with the increased abundance of intestinal beneficial bacteria *Akkermansia*, as well as the reduced abundance of opportunistic pathogenic bacteria *Campylobacterales*, *Helicobacteraceae*, and *Coriobacteriaceae*.

**Conclusions:**

The disturbed intestinal microbiota (increased abundance of opportunistic pathogens *Campylobacterales*, *Helicobacteraceae*, and *Coriobacteriaceae*) and the over‐activated TLR4/NF‐κB signaling pathway in tumor tissues deteriorated the cancer development, and the using of antibiotics is benefit to enhance the therapeutic effect of DSF on tumors via inhibiting the growth of opportunistic pathogenic bacteria.

## Graphical abstract

Abx combined with DSF/Cu^2+^ significantly inhibited tumor growth and prolonged mouse survival, via inducing the cancer apoptosis and inhibiting inflammation, enhancing the probiotics Akkermansia and Lactobacillus, and reducing the relative abundance of the pathogenic bacteria *Campylobacterales*, *Helicobacteraceae*, *Coriobacteriaceae*, and *Adlercreutzia*.

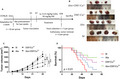

## 1 INTRODUCTION

Cancer is a group of devastating malignant diseases characterized by the abnormal cells grow beyond their usual boundaries.^1^ Cutting‐edge strategies, for example, surgery, radiotherapy, chemotherapy, and immunotherapy, have been developed for cancer treatment, while their serious side effects not only cause great suffering for patients (hair loss, anemia, nausea and vomiting, mouth ulcers and infections), but also hinder the success of cancer treatment (emergence of multidrug resistance).^2^, ^3^, ^4^ Therefore, it is a faster and cheaper alternative way to use drugs that have been approved for the treatment of multiple diseases as a candidate anticancer therapy, considering the high cost, high failure rates, and long testing periods of developing new drugs.^5^, ^6^

Disulfide is a kind of alcohol abstaining drug, which can inhibit the acetaldehyde dehydrogenase (ALDH) in cytoplasm and mitochondria, and obstruct the normal metabolism of ethanol.^7^ DSF was used in the treatment of alcohol dependence in 1948, which had good pharmacokinetics, safety and tolerability at the recommended doses of the US Food and Drug Administration (FDA).^8^ Recently, increasing evidence has indicated that DSF plays an important role in the treatment of tumors via its metabolite sodium ditiocarb (DTC, a metal chelating agent), and a stronger anti‐tumor effect is obtained when the complex of DTC and bivalent metal copper (Cu) forms.^9^, ^10^, ^11^ Previous studies have proven that DTC can bind with copper ions to form an active anticancer DTC‐copper complex (CuET) in the body, and this complex binds firmly to NPL4 (an adaptor of p97 segregase) protein in the p97‐dependent ubiquitin proteasome pathway, inhibits the garbage protein degradation function and accumulates a large number of residual proteins in cancer cells, which eventually induces the apoptosis of cancer cells.^12^ DSF is regarded as one of the promising anticancer drugs that benefit from existing clinically applicable formulations and patient tolerability, while the anti‐cancer mechanism of DSF is not fully understood.^13^

The intestinal microbiota plays an increasingly important role in cancers, diabetes, nervous system disease, and obesity.^14^, ^15^, ^16^ The relationship between intestinal microbiota and the treatment of malignant tumors is currently a research hotspot, and studies have confirmed that the intestinal microbiota plays a key role in chemotherapy and immunotherapy.^17^

Routy et al found that intestinal microbiota disorder caused the primary resistance to PD‐1 immune checkpoint inhibitors (ICIs) in patients with advanced cancer, and the use of antibiotics inhibited the clinical efficacy of ICIs, increased the relative abundance of beneficial bacteria *Akkermansia muciniphila*, which restored the efficacy of PD‐1 blockade by increasing the recruitment of CCR9^+^CXCR3^+^CD4^+^ T lymphocytes into mouse tumor beds in an Interleukin (IL)‐12–dependent manner.^18^ Chi et al indicated that intestinal microbiota regulated the growth of liver tumors by regulating the amount of natural killer T (NKT) cell in the mice liver through mediating bile acid metabolites, and the use of combined antibiotics reduced the tumor volumes and weight in mice.^19^ Moreover our studies indicated that the probiotic combination (Bifico) contained *Bifidobacterium longum*, *Lactobacillus lactis*, and *Enterococcus faecium* could significantly reduce oral mucositis caused by radiotherapy and chemotherapy in patients with nasopharyngeal carcinoma^20^; and another combination probiotics of *L. infantis*, *L. acidophilus*, *E. faecalis*, and *Bacillus cereus* significantly reduced the severity of physiological and microbial disorders induced by partial gastrectomy in patients with gastric cancer.^21^

In view of the important role of DSF and gut microbiota in cancer development and treatment, we wonder if intestinal microbes also play a role in affecting the efficacy of DSF in the treatment of cancers. Therefore, a melanoma xenograft model of C57BL/6J mice was developed in the present study, the Abx, DSF/Cu^2+^, and combination of Abx and DSF/Cu^2+^ were used to explore the potential role of intestinal microbiota in cancer treatment using DSF.

## 2 MATERIALS AND METHODS

### 2.1 Cell culture and treatment

Under aseptic conditions, B16F10 cells (Fuxiang Biological) were inoculated into a petri dish containing an appropriate amount of RPMI‐1640 medium with 10% fetal bovine serum (FBS), and cultured in an incubator at 37°C with 5% CO_2_. The medium was changed every day. When the cell coverage reached 80%~90% of the petri dish, B16F10 cells were digested with 0.25% trypsin, and then the cells were suspended and counted after washing with phosphate buffer saline (PBS), and the cell density was adjusted to prepare a cell suspension with cell density of 1 × 10^6^/mL for later use.

### 2.2 Animal studies

Seven‐week‐old female C57BL/6J mice (18‐20 g), provided by Hunan SJA Laboratory Animal, were maintained in the specific pathogen free (SPF) laboratory animal barrier system of the Institute of Translational Medicine of Nanchang University under standard conditions (humidity 51 ± 13%, temperature 23 ± 3°C, 12/12 light‐dark cycle) and were fed with standard mice maintain diet (Xietong biological, CN, Cat# 101139).

Then, 10 mice were randomly divided into C group (oral administration of 100 μL saline per day until the end of the experiment), and the remaining 64 mice were randomly divided into M group (1 × 10^5^ B16F10 cells were subcutaneously transplanted in the scapula, n = 16. Oral administration of 100 μL saline per day until the end of the experiment), Abx group (oral administration of antibiotics [ampicillin, 1 g/L; metronidazole, 1 g/L; neomycin, 1 g/L; and vancomycin, 0.5 g/L] in drinking water 2 weeks before tumor implantation until the end of the experiment, 1 × 10^5^ B16F10 cells were subcutaneously transplanted in the scapula, n = 16), DSF/Cu^2+^ group (1 × 10^5^ B16F10 cells were subcutaneously transplanted in the scapula, 50 mg/kg DSF [Sigma‐Aldrich) and 0.15 mg/kg copper gluconate [CuGlu] [Sigma‐Aldrich] on day 7 post‐tumor inoculations were administered daily until the end of the experiment, n = 16) and Abx + DSF/Cu^2+^ group (pretreated with mixed antibiotics [ampicillin, 1 g/L; metronidazole, 1 g/L; neomycin, 1 g/L; and vancomycin, 0.5 g/L] in drinking water [until the end of the experiment] for two weeks, then 1 × 10^5^ B16F10 cells were subcutaneously transplanted in the scapula, and were treated with 50 mg/kg DSF [Sigma‐Aldrich] and 0.15 mg/kg copper gluconate [CuGlu] [Sigma‐Aldrich] on day 7 post‐tumor inoculations, n = 16). Both the weight of mice and the size of tumors were measured twice per week.

At day 28th, six mice from each group (except C group) were euthanized, their tumors were removed and frozen at −80°C. The remaining mice were used to assess growth curves of tumors and survival. Tumor volume = length × width^2^ × 0.5 (repeated for three times).

### 2.3 HE staining

Tumor tissue was embedded in paraffin and sectioned about 4‐6 μm thick. The paraffin sections were dried in the oven, soaked in xylene for 10 minutes for two times, followed by gradient dehydration with ethanol. Then, sections were taken out and placed in distilled water, stained in Hematoxylin for 5 minutes, rinsed in water for 1 minute, followed with hydrochloric acid ethanol differentiation and eosin solution. After gradient dehydration by ethanol, xylene was transparent for 10 minutes for two times, sealed with neutral gum. The pathological morphology was observed under light microscope.

### 2.4 Western blotting

Appropriate amount of tumor tissue was taken into centrifuge tube, and an equal proportion of RIPA lysis buffer (Solarbio) mixed with protease inhibitor was added. After treatment with tissue homogenization on the ice, supernatant was collected via centrifuging at 12 000 g at 4°C for 10 minutes and the protein concentration was measured. Then, cellular proteins were isolated by 10%‐12% gel electrophoresis (SDS‐PAGE) and transferred to polyvinylidene fluoride (PVDF) membranes, blocked for 1 hour with 5% dry milk‐TBST (20 mM Tris‐HCl [pH 7.6], 127 mM NaCl, 0.1% Tween 20) at room temperature for 1 hour. Then PVDF was incubated overnight with appropriately diluted primary antibody at 4°C, and the secondary antibody diluted with 1% dry milk‐TBST was incubated at room temperature for 60 minutes after the washing with TBST. The following antibodies were used: rabbit anti‐ β‐actin (1:1000; Cell Signaling Technology, Cat# 4970S), mouse anti‐ TLR4 (1:1000; Santa Cruz Biotechnology, Cat# sc‐293072), rabbit anti‐ NFκB (1:1000; Cell Signaling Technology, Cat# 8242S), rabbit anti‐ p‐NFκB (1:1000; Abcam, Cat# ab86299), rabbit anti‐ B‐cell lymphoma‐2 (Bcl‐2, 1:1000; Cell Signaling Technology, Cat# 3498S), rabbit anti‐ Bcl‐2 Associated X Protein (Bax, 1:1000; Cell Signaling Technology, Cat# 14796S), rabbit anti‐ AKT (1:1000; Sangon Biotech, Cat# D151621), rabbit anti‐ p‐AKT (1:1000; Sangon Biotech, Cat# D151499).

### 2.5 Gene expression analysis

Total RNA was extracted using TRIzol Reagent (Invitrogen), and a NanoDrop 2000 spectrophotometer (Thermo Fisher Scientific) was used to determine the concentration and quality of extracted RNA. Complementary DNA (cDNA) was generated using the PrimeScript™ RT Master Mix (Takara Bio) according to the manufacturer's instructions. Quantification of gene expression was performed using a 7900HT fast real‐time PCR system (ABI) with 2 × SYBR Green master mix. Amplification was performed using the following ramping profile: 1 cycle at 95°C for 10 minutes, followed by 40 cycles of 95°C for 30 seconds, 60°C for 1 minute. The relative expression levels of IL‐6 (forward_5′‐GAAATCGTGGAAATGAG‐3′, reverse_5′‐GCTTAGGCATAACGCACT‐3′), IL‐1β (forward_5′‐GTGTCTTTCCCGTGGACCTTC‐3′, reverse_5′‐TCATCTCGGAGCCTGTAGTGC‐3′), TNF‐α (forward_5′‐GTGGAACTGGCAGAAGAGGCA‐3′, reverse_5′‐AGAGGGAGGCCATTTGGGAAC‐3′) and glyceraldehyde 3‐phosphate dehydrogenase (GAPDH; forward_5′‐CTCGTGGAGTCTACTGGTGT‐3′, reverse_5′‐GTCATCATACTTGGCAGGTT‐3′) were analysed using the 2^−ΔΔCt^ method.

### 2.6 DNA extraction and bacterial 16S rDNA sequencing

Faecal samples from groups C (N = 5), M (N = 5), Abx (N = 5), DSF/Cu^2+^ (N = 5) and Abx + DSF/Cu^2+^ (N = 5) were collected, and the bacterial genomic DNA was extracted using TIANamp Bacteria DNA Kit (TianGen) according to the manufacturer's instructions. The concentration and quality of extracted DNA were determined by NanoDrop spectrophotometer. The 16S ribosomal DNA (rDNA) V4 region was amplified using primers 515F (5ꞌ‐GTGCCAGCMGCCGCGGTAA‐3ꞌ) and 806R (5ꞌ‐GGACTACVSGGGTATCTAAT‐3ꞌ), and the PCR products were sequenced on the IlluminaHiSeq 2000 platform (Illumina, Inc).

### 2.7 High‐throughput 16S rDNA gene amplicon analysis

Paired‐end reads from the original DNA fragments were processed using Cut adapt (version 1.9.1, http://cutadapt.readthedocs.io/en/stable/) and UCHIME Algorithm (http://www.drive5.com/usearch/manual/uchime_algo.html). Sequence analysis was subsequently performed using the UPARSE software package (version 7.0.100), and sequences with ≥97% similarity were assigned to the same operational taxonomic units (OTU). Then, Qiime software (version 1.9.1) was used to analyse the α diversity (within samples, indexes of observed‐OTUs, Chao1, Shannon, Simpson, ACE and goods‐coverage) and β diversity [among samples, PCA, principle coordinates analysis (PCoA) and NMDS). Cluster analysis was preceded by weighted UniFrac distance using QIIME software (version 1.8.0), and partial least squares discriminate analysis (PLS‐DA) was performed using SIMCA‐P software version 11.5 (Umetrics; Sartorius Stedim Biotech). High‐throughput sequencing data has been uploaded to NCBI, GenBank accession number PRJNA578025.

### 2.8 Data analysis

Data analyses were performed by Prism 7 (GraphPad). Log‐rank test and One‐ or two‐way analysis of variance (ANOVA) followed by Tukey's multiple comparison test was used in all studies, as noted in figure legends. Data are presented as mean ± standard deviation (SD). Statistical significance was defined as *P < .05, **P < .01.

## 3 RESULTS

### 3.1 Abx synergistically enhanced the anticancer effects of DSF/Cu^2+^ in vivo

**FIGURE 1:**
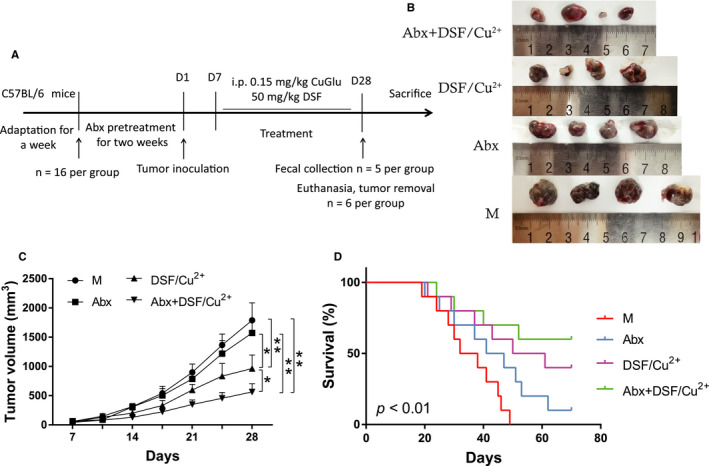
Combination of Abx and DSF/Cu^2+^ inhibited tumor growth and improved survival rate of the tumor model. (A) Treatment schedule of Abx and DSF/Cu^2+^ for mice bearing B16F10 melanoma models. (B) Images of tumor in M, Abx, DSF/Cu^2+^ and Abx + DSF/Cu^2+^ group on 28 day. (C) Changes in tumor volume over time in M, Abx, DSF/Cu^2+^ and Abx + DSF/Cu^2+^ group (N = 10). (D) Kaplan‐Meier survival curves of C57BL/6J mice melanoma xenograft models in M, Abx, DSF/Cu^2+^ and Abx + DSF/Cu^2+^ group (N = 10). Data are presented as means ± SD. Two‐way repeated‐measures ANOVA both with Tukey's test for multiple comparisons (B and C, respectively), and Log‐rank test were performed for survival data (D); *P < .05, **P < .01

To explore the effect of intestinal microbiota on the anti‐tumor drug DSF, a mice model was developed, and the anti‐cancer effects of Abx, DSF/Cu^2+^ and the combination of Abx and DSF/Cu^2+^ were evaluated (Figure 1A). Similar with previous study,^12^ the combination of Abx and DSF/Cu^2+^ showed a sound anti‐tumor effect since 17th day, and received obvious delayed tumor progression on 21st day compared with the M group (596.54 mm^3^ vs 899.99 mm^3^, *P* < .05). On 28th day, the combination of Abx and DSF/Cu^2+^ possessed the strong anti‐tumor effect compare with M group (563.23 mm^3^ vs 1788.45 mm^3^, *P* < .01), Abx group (563.23 mm^3^ vs 1572.48 mm^3^, *P* < .01) or DSF/Cu^2+^ group (563.23 mm^3^ vs 964.45 mm^3^, *P* < .05) (Figure 1B,C). The survival test indicated that all mice in M group died on 49th day, while survival rates of 10%, 40%, and 60% were obtained in Abx, DSF/Cu^2+^, and Abx + DSF/Cu^2+^ group on 70th day (Figure 1D, *P* < .01).

### 3.2 Abx + DSF/Cu^2+^ induced apoptosis and reduced inflammation in tumors

**FIGURE 2:**
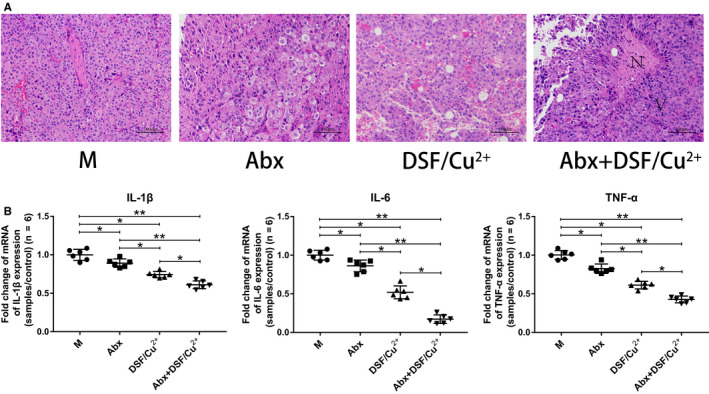
Abx enhanced the anti‐tumor effect of DSF/Cu^2+^. (A) H&E staining images of tumor tissues were presented. N: necrotic areas, V: viable tumor cells. (B) Pro‐inflammatory cytokines IL‐1β, IL‐6 and TNF‐α were detected in tumor tissues at gene level by q‐PCR. Six mice were randomly selected in each group. Data are presented as means ± SD. One‐way repeated‐measures ANOVA with Tukey's test for multiple comparisons (B); *P < .05, **P < .01

Hematoxylin‐eosin staining results indicated tumor cells in M group were dense and uniform, and the cell nucleus was deeply stained, while some necrotic tumor cells were observed in DSF/Cu^2+^ group, and large areas of nuclear pyknosis, fragmentation and dissolved necrotic areas were seen in Abx + DSF/Cu^2+^ group (Figure 2A). Furthermore, Abx, DSF/Cu^2+^ and Abx + DSF/Cu^2+^ significantly reduced the relative expressions of pro‐inflammatory cytokines IL‐1β (0.89, 0.74, and 0.61, respectively), IL‐6 (0.86, 0.52, and 0.17, respectively) and TNF‐α (0.83, 0.61, and 0.43, respectively) in tumors compared with the model group (Figure 2B; *P* < .05).

**FIGURE 3:**
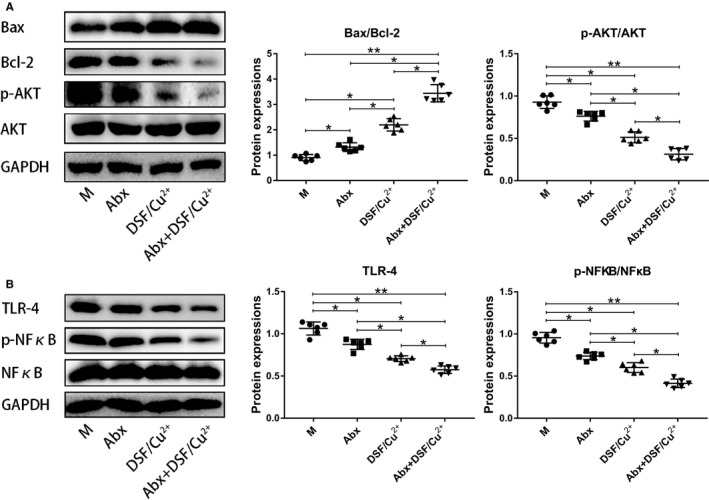
Abx synergistically enhanced the killing effect of DSF/Cu^2+^ on tumors by inducing apoptosis and reducing inflammatory response. (A) Western blot analysis of Bcl‐2, Bax, p‐AKT, and AKT expression in tumor tissues (N = 6). The relative expressions of bcl‐2, Bax, p‐akt, and AKT were quantified by Image J. (B) Western blot analysis of TLR‐4, p‐NFκB, and NFκB expression in tumor tissues, GAPDH was used as an internal control (N = 6). The relative expressions of TLR‐4, p‐NFκB, and NFκB were quantified by Image J. Data are presented as means ± SD. One‐way repeated‐measures ANOVA with Tukey's test for multiple comparisons (A and B, respectively); *P < .05, **P < .01

Then, key proteins in apoptosis pathway (Bcl‐2 family proteins) and inflammatory pathway (NFκB signal transduction) were further studied. As shown in Figure 3, Abx, DSF/Cu^2+^ and Abx + DSF/Cu^2+^ significantly enhanced the Bax/Bcl‐2 (1.32, 2.19, and 3.44, respectively), and significantly reduced the expression of p‐AKT/AKT (0.76, 0.51, and 0.31, respectively), TLR‐4 (0.87, 0.71, and 0.57, respectively), and p‐NFκB/NFκB (0.73, 0.60, and 0.41, respectively) (Figure 3A,B; *P* < .05).

### 3.3 Effect of Abx + DSF/Cu^2+^ on intestinal microbiota

**FIGURE 4:**
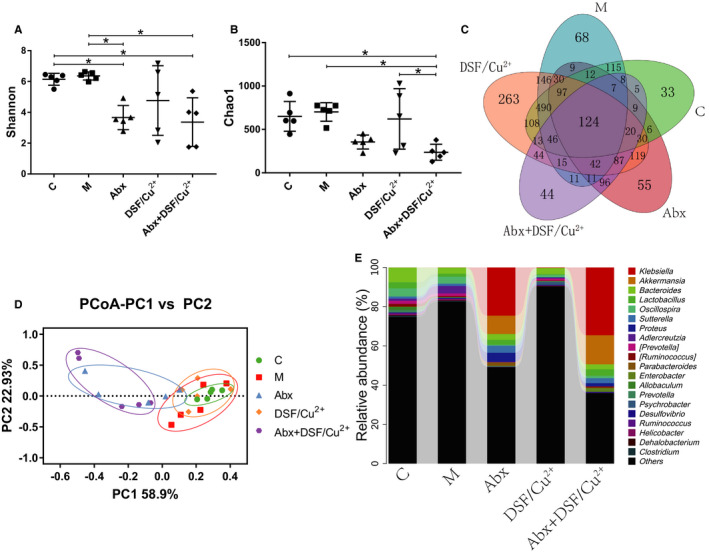
Effects of DSF/Cu^2+^ and Abx on intestinal microbiota of the tumor model. (A) The Shannon index. (B) The Chao1 index. (C) Venn map representation of OTUs. (D) PCoA of β diversity index. (E) Microbial composition at the genus level. Data are presented as means ± SD. One‐way repeated‐measures ANOVA with Tukey's test for multiple comparisons (A and B, respectively); *P < .05, **P < .01

High‐throughput sequencing method was used to study the effect of Abx, DSF/Cu^2+^ and Abx + DSF/Cu^2+^ on intestinal microbiota in melanoma xenograft mice. In total, 961 816 filtered clean tags (38 472 tags/sample), 9896 OTUs were obtained from all the samples with an average of 395.86 OTUs per group (data not shown). To better analysis the effect of Abx + DSF/Cu^2+^ on intestinal microbiota within group, the Shannon index (estimation total species) and Chao1 index (for community diversity) for alpha diversity analysis were done, and our results indicated that no obvious changes was observed between C and M group, while Abx and Abx + DSF/Cu^2+^ markedly reduced the microbial diversity and the microbial abundance compared with the C group (Figure 4A,B). When analyzed using the Venn method, 124 common OTUs were identified from all groups, and the unique OTU numbers in C, M, Abx, DSF/Cu^2+^, and Abx + DSF/Cu^2+^ were 33, 68, 55, 263, and 44, respectively (Figure 4C). PCoA analysis showed that dots were clustered in C group and relatively dispersed in M group, while DSF/Cu^2+^ treatment promotes the aggregation of dots. In addition, samples in Abx group and Abx + DSF/Cu^2+^ group had a close similarity, which was far away from the C group, meaning that the microbial diversity in Abx group and Abx + DSF/Cu^2+^ group was obvious different with that in C group (Figure 4D).

At the genus level, data of top 20 microorganism populations was analyzed. As presented in Figure 4E, *Bacteroides*, *Lactobacillus*, *Oscillospira*, *Sutterella* constituted four common dominant genus in C group (0.075 vs 0.031 vs 0.042 vs 0.011), M group (0.031 vs 0.015 vs 0.036 vs 0.012) and DSF/Cu^2+^ group (0.031 vs 0.005 vs 0.012 vs 0.001), while the treatment with Abx made *Akkermansia*, *Bacteroides*, and *Lactobacillus* as the dominant genus in Abx group (0.094 vs 0.029 vs 0.025) and Abx + DSF/Cu^2+^ group (0.148 vs 0.025 vs 0.033). Meanwhile, antibiotics obviously increased the relative abundance of opportunistic pathogenic bacteria *Klebsiella* in Abx group and Abx + DSF/Cu^2+^ group due to its strong drug resistance (Figure 4E), and the relative abundance of *Adlercreutzia* in M group were obviously increased compared with that in C group, with unknown effect on cancer.

**FIGURE 5:**
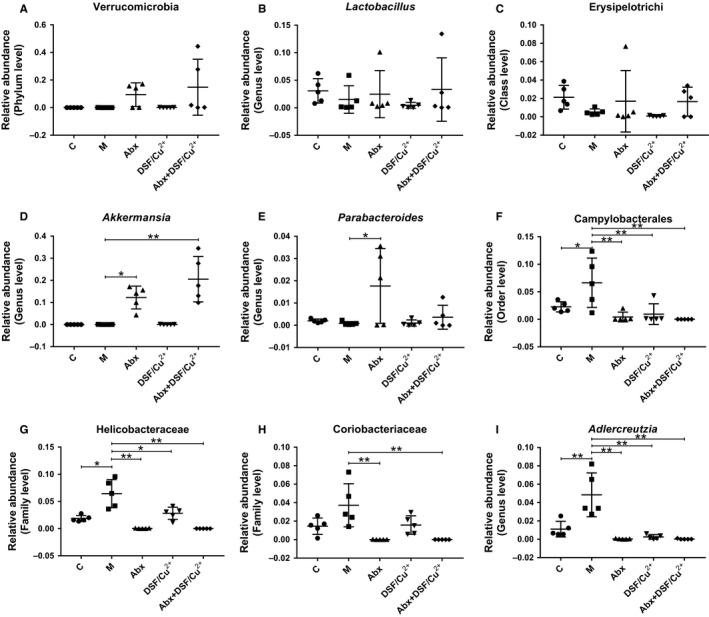
Abx synergistically enhanced the anti‐tumor effect of DSF/Cu^2+^ by increasing the abundance of intestinal beneficial bacteria. The relative abundance of *Verrucomicrobia* (A), *Lactobacillus* (B), *Erysipelotrichi* (C), *Akkermansia* (D), *Parabacteroides* (E), *Campylobacterales* (F), *Helicobacteraceae* (G), *Coriobacteriaceae* (H) and *Adlercreutzia* (I) were analyzed. Data are presented as means ± SD. One‐way repeated‐measures ANOVA with Tukey's test for multiple comparisons (A‐I, respectively); *P < .05, **P < .01

In the end, some typical bacteria closely related to cancer were chosen. The results indicated that modeling of tumor obviously increased the relative abundance of *Campylobacterales*, *Helicobacteraceae*, and *Adlercreutzia* (Figure 5F,G,I). Compared with the M group, treatment with Abx greatly increased the relative abundance of *Akkermansia* and *Parabacteroides* (Figure 5D,F), reduced the relative abundance of *Campylobacterales*, *Helicobacteraceae*, *Coriobacteriaceae*, and *Adlercreutzia* (Figure 5F‐I); treatment with Abx + DSF/Cu^2+^ greatly increased the relative abundance of *Akkermansia* (Figure 5D), reduced the relative abundance of *Campylobacterales*, *Helicobacteraceae*, *Coriobacteriaceae*, and *Adlercreutzia* (Figure 5F‐I). Interestingly, although few microbial changes in *Verrucomicrobia*, *Lactobacillus*, *Erysipelotrichi*, *Akkermansia*, and *Parabacteroides* were observed between M group and DSF/Cu^2+^ group (Figure 5A‐E), DSF/Cu^2+^ obviously reduced the relative abundance of *Campylobacterales*, *Helicobacteraceae*, and *Adlercreutzia* (Figure 5F,G,I).

## 4 DISCUSSION

The high incidence rate and the resistant of cancers to current available drugs make it is an inevitable choice to develop anti‐tumor drugs.^22^, ^23^ However, it is a good choice to expand new anti‐tumor features from traditional non‐neoplastic drugs, considering the long time, big investment and high failure rate of developing new drugs.^24^ In recent years, Metformin (a classic drug for the treatment of diabetes) and Aspirin (most common antipyretic analgesic anti‐rheumatic drug) have been expanded to cancers,^25^ cardiovascular disease and colorectal cancer,^26^ respectively. Moreover DSF was reported to reduce the mortality rate of cancer patients by 34% in a recent study.^12^, ^27^

With the development of molecular biology technology, more and more studies verified the role of intestinal microbiota on therapeutic effect of anti‐tumor drugs.^28^ As an oral drug, whether the DSF can affect the intestinal microbial diversity of host, or if the intestinal microbiota can affect the therapeutic effect of DSF? In the present study, a melanoma xenograft model was developed to answer these questions, which deepened the understanding of DSF and intestinal microbiota, and provided data to support the clinical application of DSF.

Consistent with previous researches, our results indicated that using of DSF/Cu^2+^ alone could delay tumor progression and prolong survival of mice, while the combination of Abx pretreatment and DSF/Cu^2+^ significantly inhibited tumor growth and greatly improved survival rate and extended life span of mice (Figure 1). Interestingly, mice treated with Abx alone also showed a visible reduction in tumor size. The HE staining and qPCR results indicated that DSF/Cu^2+^ resulted in necrotic areas of tumor tissues, and Abx could synergistically increase tumor necrosis areas to DSF/Cu^2+^, and the overexpression of pro‐inflammatory cytokines IL‐1β, IL‐6, and TNF‐α were observed in model group, while the combination of Abx and DSF/Cu^2+^ could reverse the level of inflammation (Figure 2). DSF is an anti‐tumor drug newly discovered from many old drugs. Studies have shown that DSF is metabolized into DTC and combined with Cu^2+^ to form a new compound CuET in the body, which can inhibit tumor growth by blocking the degradation pathway of the waste protein in the tumor cells and cause apoptosis of the tumor cells,^12^ conformed by our study.

Many studies indicated that antibiotics could inhibit cancer or affect the treatment of cancer, while the sustained inflammatory response will form tumor microenvironment with a large number of immunosuppressive cells such as myeloid‐derived suppressor cells (MDSCs), regulatory T cells and tumor‐associated suppressor cells, to promote tumor immune escape, tumor growth and metastasis. In 2018, Sethi et al found that gut microbiome depletion by oral antibiotics significantly inhibited tumor growth in subcutaneous and liver metastasis models of pancreatic cancer, colon cancer, and melanoma.^29^ Similarly, our work also indicated that Abx combined with the anti‐tumor drug DSF/Cu^2+^ had significantly inhibited the tumor growth in the melanoma‐bearing mice, accompanied by the reducing of inflammatory cytokines IL‐1β, IL‐6, and TNF‐α.

To further study the potential mechanisms of DSF/Cu^2+^ on cancers, we studied the key proteins in inflammation, growth and apoptosis signaling pathways. It is well known that Bcl‐2 and Bax are members of the Bcl‐2 gene family, among which Bcl‐2 is particularly considered as an important anti‐apoptotic protein, while Bax is up‐regulated by tumor suppressor protein p53 and participates in p53‐mediated apoptosis.^30^ Bcl‐2 and Bax can regulate tumor cell apoptosis by regulating Caspase protein activity. In most tumors, Bcl‐2 expression levels are elevated, while Bax expression levels are reduced, which leads to an increase in the amount of Bcl‐2/Bax heterodimers and Bcl‐2 homodimers, thereby inhibiting tumor cell death.^30^ As a key protein in growth, AKT is an important apoptotic inhibitor in vivo, which induces the expression of various genes encoding anti‐apoptotic proteins,^31^ meanwhile, p‐AKT can directly regulate the formation of Bcl‐2/Bax heterodimer through PI3K‐Akt signaling pathway and inhibit tumor cell apoptosis.^32^ In addition, TLR4/NF‐κB signaling pathway plays an important role in cancer development. NF‐κB is an important regulator in immune and inflammatory processes, which can induce the expression of various pro‐inflammatory factors through TLR‐MyD88 signaling pathway to enhance the survival of tumor cells and promote their proliferation.^33^ Therefore, the significant increase of Bax/Bcl‐2, and the significant reduction in p‐AKT/AKT and TLR4/NF‐κB indicated that combined treatment with Abx and DSF/Cu^2+^ significantly attenuates inflammatory effects and promoted the apoptosis of cancer cells (Figure 3).

In the end, high‐throughput sequencing method was used to monitor the microbial changes in host intestines. It seemed that DSF/Cu^2+^ promoted the transformation of intestinal microbiota in tumor mice to control mice, while the treatment with Abx and DSF/Cu^2+^ obviously changed the microbial compositions, and lowered the bacterial abundance (Figure 4). Moreover studies have shown that the relative abundance of *Verrucomicrobia* and *Akkermansia* reduced in the feces of patients when received broad‐spectrum antibiotics, because antibiotics killed most of the bacteria in the intestine, and *Akkermansia* showed resistance to vancomycin and metronidazole.^34^ *Akkermansia* belongs to *Verrucomicrobia*, which was identified by scientists in 2004 and can use intestinal mucin as an energy source to protect the intestine from pathogens through competitive action.^35^ A number of studies have shown that a reduced abundance of *Akkermansia* had been observed in patients with inflammatory bowel disease, obesity, and type 2 diabetes, and supplementation with *Akkermansia* could improve these symptoms.^36^, ^37^ In addition, recent studies have shown that *Lactobacillus* or *Akkermansia* combined with anticancer drugs can significantly inhibit tumor growth,^38^ while the relative abundance of *Helicobacteraceae*, *Campylobacter* in the intestine can induce tumor development.^39^ Therefore, the increased abundance of intestinal beneficial bacteria *Akkermansia*, and the reduced abundance of *Campylobacterales*, *Helicobacteraceae*, and *Adlercreutzia* in Abx and DSF/Cu^2+^ confirmed their anti‐cancer effects (Figures 4 and 5).

Taken together, our results indicated that Abx combined with DSF/Cu^2+^ significantly inhibited tumor growth and prolonged mouse survival, via inducing the cancer apoptosis and inhibiting inflammation, enhancing the intestinal beneficial bacteria *Akkermansia*, and reducing the relative abundance of the opportunistic pathogenic bacteria *Campylobacterales*, *Helicobacteraceae*, and *Coriobacteriaceae*. In the present study, we firstly explore the potential effect of intestinal microbiota when treated cancer using DSF, providing data on its potential use in clinic.

## CONFLICT OF INTEREST

The authors declare that there are no competing interests regarding the publication of this paper and regarding the funding that they have received.

## AUTHOR CONTRIBUTIONS

Tingtao Chen, Shaobo Li, and Chuangwei Zou designed the study. Hong Hu, Tingtao Chen, Lanyue Cui, Jiachen Lu, and Jing Wei carried out the experiments and analyzed the references and wrote the manuscript. All authors discussed the results and commented on the manuscript.

## ETHICAL APPROVAL

The animal study was approved by the ethics committee of the second affiliated hospital of Nanchang University, and all the methods were carried out in accordance with the approved guidelines.

## Data Availability

The datasets used and/or analyzed during the current study are available from the corresponding author on reasonable request.
